# MiR-365-3p is a negative regulator in IL-17-mediated asthmatic inflammation

**DOI:** 10.3389/fimmu.2022.953714

**Published:** 2022-07-26

**Authors:** Weijia Wang, Ying Li, Jiaqi Fan, Xiaoyan Qu, Dong Shang, Qiaohong Qin, Tun Xu, Qutayba Hamid, Xiaomin Dang, Ying Chang, Dan Xu

**Affiliations:** ^1^ The Key Laboratory of Biomedical Information Engineering of Ministry of Education, School of Life Science and Technology, Xi’an Jiaotong University, Xi’an, China; ^2^ Department of Respiration, The First Affiliated Hospital, Xi’an Jiaotong University, Xi’an, China; ^3^ Institute of Basic and Translational Medicine, Xi’an Medical University, Xi’an, China; ^4^ School of Automation Science and Engineering, Faculty of Electronic and Information Engineering, Xi’an Jiaotong University, Xi’an, China; ^5^ Meakins-Christie Laboratories and Respiratory Division, The Research Institute of the McGill University Health Centre and Department of Medicine, McGill University, Montreal, QC, Canada; ^6^ College of Medicine, University of Sharjah, Sharjah, United Arab Emirates

**Keywords:** asthma, IL-17KO, miR-365-3p, ARRB2, IL-8

## Abstract

**Background:**

Interleukin-17, the major proinflammatory cytokine secreted by Th17 cells, makes essential contribution to pathogenesis of severe asthma, while the detailed mechanisms, especially the involvement of microRNAs which are also important participants in asthma progression, remains largely unclear.

**Methods:**

In this study, we established a house dust mite (HDM) extract-induced murine asthmatic models and the miRNA expression in the lung tissues of mice were profiled by miRNA microarray assay. The effect of miR-365-3p on IL-17-mediated inflammation was examined by qRT-PCR and immunoblotting analysis. The involvement of ARRB2 as target gene of miR-365-3p was verified by overexpression or RNA interference.

**Results:**

HDM extract-induced asthmatic inflammation was proved to be IL17-mediated and miR-365-3p was screened out to be the only miRNA exclusively responsive to IL-17. miR-365-3p, whose expression was significantly downregulated upon IL-17 stimulation, was demonstrated to exert remarkable anti-inflammatory effect to decrease IL-17-provoked inflammatory cytokines (KC/IL-8 and IL-6) in both airway epithelial cells and macrophages of murine and human origins, verifying its universal antagonizing activity against IL-17-initiated inflammation across the two species. ARRB2 was characterized as the key target of miR-365-3p to negate IL-17-induced inflammatory cytokines.

**Conclusion:**

Taken together, our data supported the notion that miR-365-3p, which was diminished by IL-17 in murine and human asthmatic pathogenesis, functioned as an essential negative mediator in IL-17-stimuated inflammatory response by targeting ARRB2, which would shed new light to the understanding and therapeutics thereof of asthmatic inflammation.

## Background

Asthma is a chronic inflammatory airway disease characterized by reversible airflow limitation with complicated pathogenesis involving variety of inflammatory cells and cytokines (1), of which Th17 cells, the crucial mediators between innate immunity and adaptive immunity, are shown to play essential roles in the inflammatory response in asthma (2). Our previous studies found that cytokines secreted by Th17 cells can induce the chemotaxis, proliferation and inflammatory responses of airway smooth muscle cells (ASMCs), revealing the unneglectable participation of Th17 cytokines in the remodeling of airway (3–5). IL-17 (also called IL-17A), the main contributor of the pro-inflammatory activity of Th17 cells (2, 6) has been demonstrated to be positively correlated with severity and steroid insensitivity of asthma (7, 8), whereas the detailed mechanisms remain to be explored.

MicroRNAs, the prominent regulators of gene expression by targeting mRNAs for degradation or translational suppression (9), also actively participate in the airway inflammatory response, as exampled by miR-21 that was identified as an essential regulator in Th1/Th2 imbalance and subsequent AHR, mucus formation and eosinophil infiltration in mice airway inflammation (10), miR34/449 that was suppressed by IL-13 to promote bronchial epithelial cells differentiation and proliferation (11), and miR-19a and miR-221 that played an essential role in proliferation of epithelial cells and airway smooth muscle cells in severe asthmatic patients (12, 13). Although the role of miRNA has been well recognized in asthma, its involvement in IL17-mediated asthmatic inflammation has not been fully understood (9, 14, 15). Nonetheless, a recent study revealed that miRNA was indispensable for IL-17 to induce secretion of inflammatory factors and chemokines by astrocytes in experimental autoimmune encephalomyelitis (16), implying the possibility that miRNA also participates in IL-17 -mediated asthmatic pathogenesis.

To investigate the role of miRNA in IL-17-dependent asthmatic inflammatory response, a chronic asthmatic animal model was established in wild type and IL-17- deficient mice. miRNA profiling assays and analysis revealed that miR-365-3p was significantly downregulated by IL-17. The further mechanistic study demonstrated that miR-365-3p was a negative regulator in IL17-mediated inflammation in both murine and human airway cells *via* miR-365-3p/ARRB2/cytokines axis, which may provide experimental evidence for the potential therapeutic targets of asthma.

## Methods and materials

### Animals and establishment of murine asthmatic models

C57BL/6 female mice (6-8 wk) were purchased from Charles River (Montreal, Canada). IL-17KO (IL-17A deficient) mice were prepared as previously described (17). All animals were treated and maintained under a protocol approved by the Animal Care Committee of McGill University, following guidelines set by the Canadian Council on Animal Use and Care. To establish asthmatic model, wild type and IL-17KO mice were exposed to purified home dust mite (HDM) extract (Greer Laboratories, Lenoir, NC) intranasally (25 μg of protein in 10 μl of saline) for 5 days/week for five consecutive weeks. The equivalent volume of saline was used as the control. Each group contained 10 mice. The mice were sacrificed 24 h after the last exposure.

### Preparation of bronchoalveolar lavage fluid

The lungs were lavaged using a cannula inserted in the trachea and the lungs were instilled with 0.5 ml PBS. Cytospins were prepared at a density of 0.5 × 10^6^ cells/ml. Differential cell counts were performed using standard morphological criteria on Hema-Gurr-stained cytospins (500 cells/sample) (Merck, Darmstadt, Germany).

### Bronchoalveolar lavage cytokine analysis

Aliquots of cell-free bronchoalveolar lavage fluid (BALF) were frozen in liquid N_2_ and stored at -80°C. The levels of IL-17, IL-4, IL-5, IL-13 and KC in BALF were analyzed by the Bio-plex system (Bio-Rad, Mississauga, Ontario, Canada) performed in the Goodman Cancer Centre Transgenic Core Facility, McGill University, Canada.

### Immunohistochemistry staining

The immunohistochemistry staining for IL-17A (Santa Cruz Biotechnology, Santa Cruz, CA, USA) and α-SMA (Santa Cruz Biotechnology) was performed on paraffin-embedded mouse lung tissue sections as previously described (18).

### miRNA expression array

Equal amount of RNA sample from each mouse of different groups was pooled individually for miRNA profiling assay using µParaflo^®^Microfluidic Biochip miRNA microarray (LC Sciences, Houston, USA).

### Real-time quantitative RT-PCR

Three independent assays were performed using quantitative reverse transcription PCR (qRT-PCR) on all mouse samples for each individual miRNA (miR-207, miR-5112, miR-2861, miR-340-5p, miR-6238, miR-181c-5p, miR-6239, miR-365-3p and miR-133b-3p) (Qiagen, Hilden, Germany) and predicted target genes (FASL, TRAF3, ARRB2, Sgk1, PIK3R3, ADAM10 and ADM) (Bio-rad, Foster City, USA). The inflammatory cytokines expression in MLE-12, J774a.1, A549 and U937 cells were analyzed by qRT-PCR as well. Data were presented relative to U6 for miRNA and β-actin for target genes based on calculations of 2^(−σCt)^. The primer sequences for target genes were listed in **Table 1**. Statistical significance was defined as p < 0.05 as measured by the t-test using GraphPad Prism 5 software (GraphPad, San Diego, USA).

**Table 1:** The sequence of primers for real-time PCR.

Gene	Sequence (5’-3’)	Direction
FASL	GAACTCCGTGAGCCAACCC	Forward
	CCAGAGATCAGAGCGGTTCC	Reverse
TRAF3	GCGTGCCAAGAAAGCATCAT	Forward
	CCTCTGCCTTCATTCCGACA	Reverse
ARRB2	ACACGCCACTTCCTCATGTC	Forward
	TCTTCTTGACGGTCTTGGCA	Reverse
Sgk1	ATCCTGACCAAGCCGGACC	Forward
	AAAATCGTTCAGGCCCATCCTT	Reverse
PIK3R3	AAGATGCAGAGTGGTACTGGG	Forward
	CCTGCATTTTCGTTGAGGCA	Reverse
ADAM10	ACACCAAAAACACCAGCGTG	Forward
	GGAAGTGTCCCTCTTCATTCGT	Reverse
ADM	ATTGGGTTCACTCGCTTTCCT	Forward
	GCTGGATGCTTGTAGTTCCCT	Reverse
KC	GCTTGAAGGTGTTGCCCTCAG	Forward
	AAGCCTCGCGACCATTCTTG	Reverse
Mouse IL-6	ACAAAGCCAGAGTCCTTCAGAG	Forward
	AGGAGAGCATTGGAAATTGGGG	Reverse
Mouse β-actin	GATGCCCTGAGGCTCTTTTCC	Forward
	TCTTTACGGATGTCAACGTCACAC	Reverse
IL-8	GATGCCAGTGAAACTTCAAGC	Forward
	ATTGCATCTGGCAACCCTAC	Reverse
Human IL-6	CCAGAGCTGTGCAGATGAGT	Forward
	ATTTGTGGTTGGGTCAGGGG	Reverse
Human ARRB2	TGTGTCTGGGGTGGGGATAC	Forward
	AGCCGCACAGAGTTCCTTTT	Reverse
Human β-actin	TGGCACCCAGCACAATGAA	Forward
	CTAAGTCATAGTCCGCCTAGAAGCA	Reverse

### Cell culture

MLE-12, 293T, J774a.1, U937 and A549 cell lines were purchased from ATCC (Manassas, VA). MLE-12 were cultured in HITES medium (50:50, DMEM-Ham’s F-12) supplemented with 2% FBS, 1:100 insulin/transferrin/selenium supplement. 293T and J774a.1 was maintained with high glycose DMEM with 10% FBS, while U937 and A549 were maintained in 1640 medium with 10% FBS. All the cells culture medium was supplemented with penicillin (100 U/ml) and streptomycin (100 μg/ml). Before the transfection, U937 was differentiated into macrophage-like cells by 72h stimulation with 200nM PMA (phorbol 12-myristate 13-acetate). HITES medium were purchased from Hyclone (Logan, UT, USA), while DMEM and 1640 medium were provided by Gibco (Grand Island, NY, USA). ITS supplement 100X, penicillin/streptomycin 100X and PMA were supplied by Sigma (St. Louis, MO, USA).

### MiR-365-3p transfection

After 24 h of starvation with HITES medium containing 0.1% FBS, MLE-12 cells were cultured with different concentrations (0, 1, 10 and 100ng/ml) of mouse recombinant murine IL-17A (R&D Systems, Minneapolis, USA) for 24h, and the expression of miR-365-3p was examined by qRT-PCR. Alternatively, the mimic (100nM) of miR-365-3p was transfected into the MLE-12 cells using X-tremeGENE siRNA Transfection Regent (Roche, Penzberg, Upper Bavaria, Germany) in Opti-MEM (Gibco) for 24h. Expression of ARRB2 was examined by qRT-PCR and Western blot.

We cultured 4 kinds of cells for MiR-365-3p transfection, including MLE-12, J774a.1, U937 and A549. the cells were transfected with the mimic (100nM) of miR-365-3p for 6h, and 24h later, the transfected cells were incubated with 10ng/ml murine IL-17A or 100 ng/ml human IL-17A (R&D Systems). RNA samples were collected 3h after the IL-17A stimulation. qRT-PCR were performed to evaluate the level of cytokines in IL-17A treated cells.

### Luciferase reporter assay

Sense and antisense sequences corresponding to a 406-bp fragment from the 3’UTR of ARRB2 with the predicted binding and mutated sites (position 236–243) were amplified from cDNAs of mouse lung tissue by using primers containing the *SacI* restriction site in the sense oligo and *XbaI* restriction site in the antisense oligo (Sense: 5’-ATCGAGCTCCTGTCCACCCGAGATACAC-3’; Antisense: 5’-AGCTCTAGAGGTACCCTGCAGATGTAGAA-3’; GenePharma, Shanghai, China). To construct luciferase reporter plasmids for ARRB2, the annealed synthetic oligos were cloned downstream to the firefly luciferase into *SacI-XbaI* double digested pmirGLO Dual-Luciferase miRNA target expression vector (Promega, WI, USA).

For the luciferase reporter assay, 293T cells were co-transfected with 250 ng of luciferase reporter plasmid harboring the wild type/mutant binding sites of ARRB2 respectively along with 25nM mimic control/miR-365-3p mimic using X-tremeGENE siRNA Transfection Regent (Roche) in Opti-MEM (Gibco). After 48h of transfection, cells were washed in PBS and lysed in Reporter lysis buffer (Promega), and luciferase activity was measured in a FlexStation 3 microplate reader (Molecular Devices, Sunnyvale, CA, USA) using the Dual-Luciferase reporter assay kit (Promega) according to the manufacturer’s instructions. Firefly luciferase activity was normalized to Renilla luciferase activity, and relative luciferase activity was calculated taking firefly luciferase activity of empty pmirGLO transfected cells as 100 percent.

### ARRB2 overexpression and knockdown assay

Mouse *ARRB2* mRNA was cloned from cDNA of mouse lung tissue by PCR, the primers containing *NheI* restriction site in the sense oligo and *HindIII* restriction site in the antisense oligo (Sense: 5’- CTAGCTAGCATGGGAGAAAAACCCGGGAC -3’; Antisense: 5’- CAGAAGCTTCTAGCAAAACTGGTCATCACAGTC-3’; TsingKe, Beijing, China). To construct ARRB2-overexpression vector, the gene were cloned into the multiple cloning site of *NheI- HindIII* double digested pcDNA3.1 plasmid (Invitrogen, CA, USA). To knock down the expression of ARRB2, the siRNA was purchased from TsingKe (Sense: 5’- GGACCAGGGUCUUCAAGAATT -3’; Antisense: 5’- UUCUUGAAGACCCUGGUCCTT-3’). For the ARRB2 overexpression or knockdown assay, the pcDNA3.1-ARRB2 plasmid (1μg) and ARRB2-siRNA (130ng) were transfected into MLE-12 cells by X-tremeGENE siRNA Transfection Regent (Roche) in Opti-MEM (Gibco). After 24h transfection, the transfected cells were treated with IL-17 (10ng/ml) for 3 hours and examined by qRT-PCR and Western blot.

### Western blot

The protein samples of mouse lung tissue or MLE-12 cells were loaded (5 μg) on a 10% acrylamide SDS-PAGE gel (Bio-Rad, Hercules, USA) for protein separation, followed by transfer to PVDF membranes (Bio-Rad). The blots were then blocked with 1% BSA in 0.1% Tween 20/TBS for 1 h at room temperature and then incubated overnight at 4°C with antibodies specific for ARRB2 (Novus Biologicals, Centennial, CO, USA). After washing with 0.1% Tween 20 in TBS, the membranes were incubated with a 1: 3000 dilution of goat anti-rabbit IgG HRP (EMD Millipore Corp, Burlington, Massachusetts, USA) in 1% solution of powdered milk in TBS/0.1% Tween 20. The membranes were exposed to ECL solution (Bio-Rad) and imaged by chemiluminescence (Clinx Science Instrument, Shanghai, China).

### Statistical analysis

**Figure 1:**
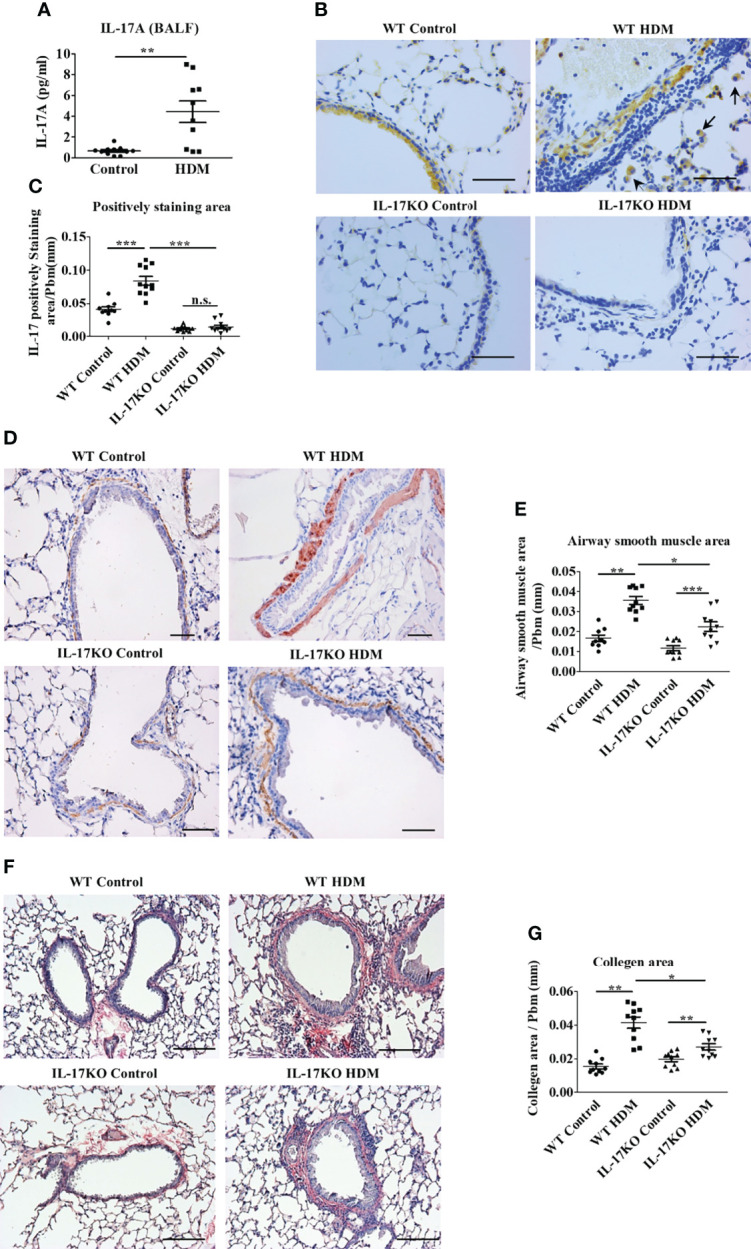
IL-17 is necessary to maintain asthmatic phenotype in HDM-induced murine model. (**A)** The level of IL-17A in mouse Bronchoalveolar lavage fluid (BALF) detected by Bio-Plex. N=10. **(B, C)** IHC staining **(B)** and quantitative analysis **(C)** of IL-17 positive cells in mouse lung tissue section. The brown area represents IL-17 positive. Black arrows indicate stained macrophage. Scale bar =50μm. N=10. **(D, E)** IHC staining **(D)** and quantitative analysis **(E)** of airway smooth muscle mass (indicated by Alpha-Smooth Muscle Actin) in mouse lung tissue section. The brown areas represent the airway smooth muscle cells. Scale bars= 100μm. N=10. **(F, G)** collagen deposition analysis in mouse lung tissue. Collagen was stained by Picro Sirius red, the color red indicates the positive staining. Scale bars=100μm. N=10. *, P<0.05, **, P<0.01, ***, P<0.001. n.s., not statistically significant.

**Figure 2:**
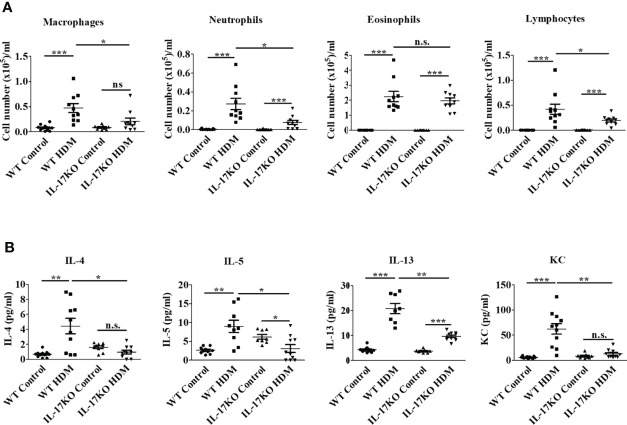
Indispensable role of IL-17 in the inflammatory response of HDM-induced asthma in murine model. **(A)** Counts of inflammatory cells in BALF. N=10. **(B)** The level of inflammatory cytokines in BALF. The inflammatory cytokines in BALF were detected by Bio-Plex. N=10. *, P<0.05, **,P<0.01, ***, P<0.001. n.s., not statistically significant.

**Figure 3:**
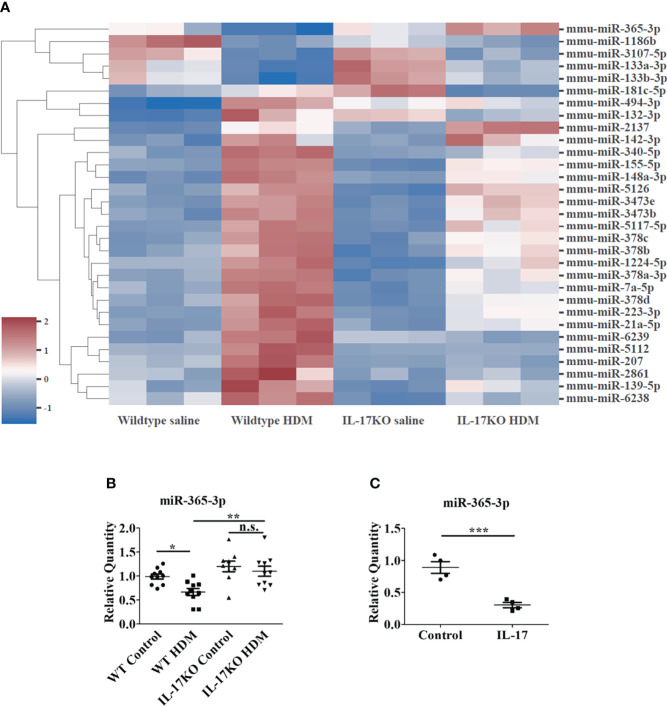
miR-365-3p is downregulated in wildtype HDM-induced mice but not in IL-17 KO HDM-induced mice. **(A)** Microarray of dysregulated miRNA in wildtype saline and HDM-induced mice, N=3. **(B)** The level of miR-365-3p examined by qRT-PCR in wild-type and IL-17KO mice. N=10. **(C)** The effect of IL-17 in miR-365-3p expression in MLE-12 cells. The MLE-12 cells were treated by IL-17, and the miR-365-3p expression was tested by qRT-PCR N=4. *, P<0.05, **, P<0.01, ***, P<0.001. n.s., not statistically significant.

**Figure 4:**
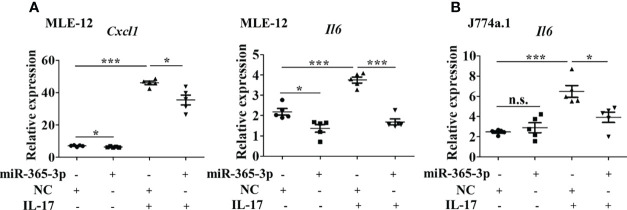
miR-365-3p negates IL-17-induced inflammatory cytokines expression in murine epithelial cells and macrophages. **(A)** Effect of miR-365-3p mimic on mRNA expression of KC and IL-6 in MLE-12 cells upon IL-17 treatment. N=5. **(B)** Effect of miR-365-3p mimic on mRNA expression of IL-6 in J774a.1 cells upon IL-17 treatment. N=3. *, P<0.05, ***, P<0.001. n.s., not statistically significant.

**Figure 5:**
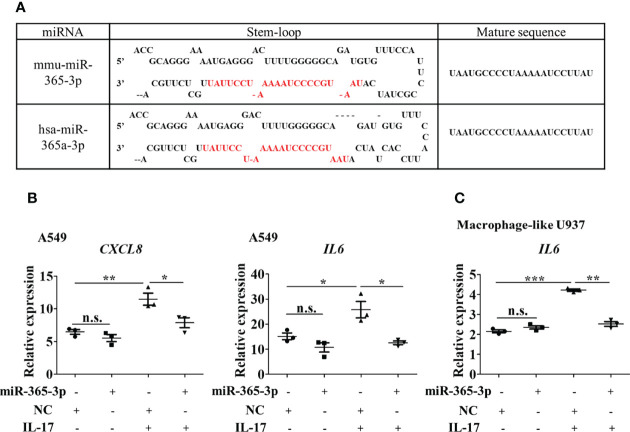
miR-365-3p also serves as a negative regulator in hIL-17 induced inflammatory response in human airway epithelial cells and macrophages. **(A)** Sequence comparison of mmu-miR-365-3p and has-miR-365a-3p. **(B)** Effect of miR-365-3p mimic on mRNA expression of IL-8 and IL-6 in human A549 cells upon hIL-17 treatment. N=3. **(C)** Effect of miR-365-3p mimic on mRNA expression of IL-6 in PMA-differentiated U937 cells upon hIL-17 treatment. N=3. *, P<0.05, **,P<0.01, ***, P<0.001. n.s., not statistically significant.

**Figure 6:**
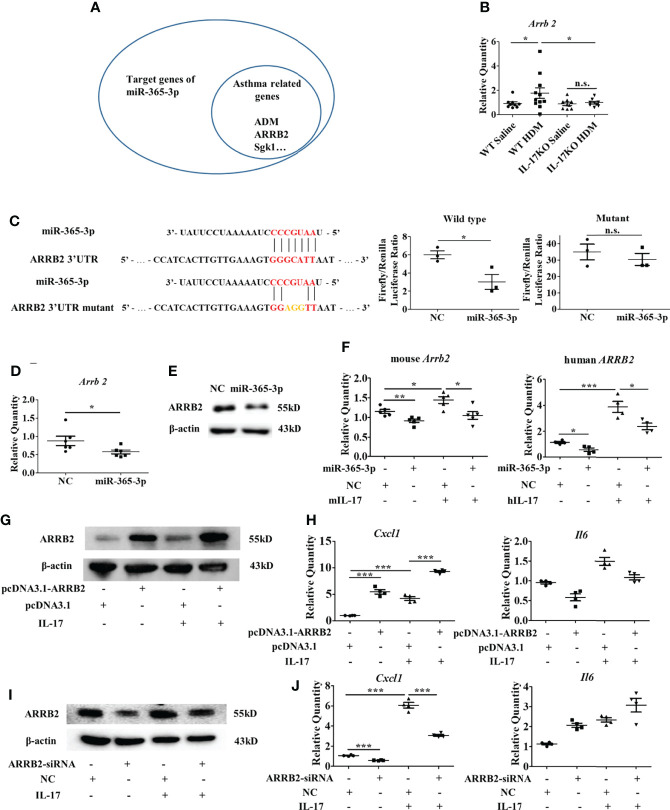
ARRB2 is the target gene of miR-365-3p to negate proinflammatory effect of IL-17. **(A)** target genes of miR-365-3p that are related with asthma. **(B)** mRNA expression of *A*RRB2 detected by qRT-PCR. N=5. **(C)** The targeting role of miR-365-3p on *A*RRB2 examined by reporter fluorescence assays, N=3. **(D)** Effect of miR-365-3p rescue on mRNA level of *A*RRB2. N=6. **(E)** Effect of miR-365-3p rescue on protein level of ARRB2. **(F)** regulatory effect of miR-365-3p on ARRB2 expression in both mouse MLE-12 (left) and human A549 (right) epithelial cells. **(G)** immunoblotting analysis of ARRB2 overexpression from pcDNA3.1 vector in MLE-12 cells. **(H)** expression of KC and IL-6 in ARRB2-overexpression MLE-12 cells upon IL-17 treatment. N=4. **(I)** immunoblotting analysis of ARRB2 knockdown by siRNA in MLE-12 cells. **(J)** expression of KC and IL-6 in ARRB2-knockdown MLE-12 cells upon IL-17 treatment. N=4. *, P<0.05, **, P<0.01, ***, P<0.001. n.s., not statistically significant.

Results are expressed as means ± SE. Statistical analysis for expression of IL-17A in mouse lung tissue (**Figure 1A**) was performed by Mann-Whitney test. The differences between the four groups of mice (**Figures 1**, **2**, **3B**, **6B** and **Supplementary Figures 1**, **2**) were evaluated by Kruskal-Wallis test. The results of cell culture experiments (**Figures 3C**, **4**, **5**, **6C–H**) were assessed by t-test. Probability values of P < 0. 05 were considered significant. Data analysis was performed by using the GraphPad Prism 5 software (GraphPad, San Diego, CA, USA).

## Results

### IL-17 is indispensable for asthmatic phenotype in HDM-induced murine model

To examine the alteration of IL-17 in asthmatic pathogenesis, a murine model was generated by repeated intranasal challenge with house dust mite extract (HDM) 5 times a week for 5 weeks. The level of IL-17 in bronchoalveolar lavage fluid (BALF) in the asthmatic group were 4-fold higher than control group (P<0.01, **Figure 1A**), which was further confirmed by the elevated proportion of IL-17-positive areas in the lung tissue of asthmatic animals as shown by immunohistochemical analysis (**Figures 1B** and **1C**). According to their morphology, most of the IL-17 expressing cells were categorized as macrophages (19) and lymphocytes. The indispensability of IL-17 to maintain asthmatic phenotype was then verified in IL-17KO mice, which developed significantly less pronounced symptoms upon induction of HDM extract as evidenced by thinner airway wall and reduced inflammatory cell infiltration compared with wild-type asthmatic mice (**Supplementary Figure 1**). As a typical asthmatic histopathological feature, the thickened layer of smooth muscle was much more prominent in wild-type asthmatic model than IL-17KO asthmatic model, as shown in immunohistochemical staining (**Figures 1D, E**), which coincided with the distinguishable collagen deposition between the lung tissues of wildtype and IL-17KO asthmatic model (**Figures 1F, G**).

### IL-17 is pivotal for the inflammatory response in murine asthmatic model

To further elucidate the role of IL-17 in the inflammatory response in the murine asthmatic model, inflammatory cells of different types in BALF were firstly numerated, which showed that wildtype asthmatic mice had significantly higher number of inflammatory cells including macrophages, neutrophils and lymphocytes in their BALF than mock treatment (**Figure 2A** and **Supplementary Figure 2**). In stark contrast, inflammatory cells in the BALF of mice with IL-17 deficiency were significantly less than wildtype counterparts upon HDM stimulation (**Figure 2A**), indicating the IL-17 was the main contributor to increase inflammatory cells in BALF during asthmatic pathogenesis.

Moreover, the Th2 cytokines in BALF of HDM-stimulated wild-type mice increased by 6.6-fold for IL-4, 3.4-fold for IL-5 and 4.8-fold for IL-13, respectively, along with 10.6-fold elevation of pro-inflammatory cytokine KC (human IL-8 homologue), which was believed to be the main effector cytokine to recruit neutrophils in asthma. Nevertheless, inflammatory response represented by these cytokines was largely abolished in IL-17KO asthmatic model animals (**Figure 2B**). The level of IFN-gamma did not show obvious alterations upon HDM treatment in both wildtype and IL-17KO mice, indicating that IFN-gamma did not play a significant role in this asthmatic model (**Supplementary Figure 3**). Collectively, our data implied that IL-17 was pivotal for the inflammatory response in HDM-induced murine asthma model.

### miR-365-3p is downregulated in wildtype asthmatic mice but not in IL-17 KO asthmatic mice

To investigate the miRNAs involved in IL-17-dominated asthmatic pathogenesis, the miRNA expression in the lung tissues of mice were profiled by miRNA microarray assay. The results showed that the expression of 31 miRNAs in WT model mice changed by more than 1.5 times compared with WT control group (**Figure 3A**), of which 9 were selected for the further analysis due to their exclusive responsiveness to IL-17 since they were not altered between IL-17KO model and IL-17KO control mice. We further verified the expressions of the 9 miRNAs in mouse lung tissue by qRT-PCR (**Supplementary Figure 4**), which confirmed that only miR-340-5p and miR-365-3p were significantly different between wildtype control and wildtype asthmatic group, whereas miR-365-3p is the only one that was exclusively responsive to IL-17 (**Figure 3B**). The negative regulatory effect of IL-17 on miR-365-3p was further confirmed by a ~90% reduction of expression level upon treatment with 10 ng/mL IL-17 in mouse alveolar epithelial cells (MLE-12) (**Figure 3C**).

### IL-17 induces inflammatory response through downregulating miR-365-3p

Since miR-365-3p was the only miRNA selected to be responsive to IL-17, we next, logically, investigated the mechanisms how miR-365-3p participated in IL-17-mediated inflammatory response in asthma. Both mouse alveolar epithelial cells (MLE-12) and macrophages (J774a.1) were used to examine the role of miR-365-3p in IL-17-mediated inflammation. We then examined the influence of miR-365-3p on the pro-inflammatory activity of IL-17 by transfecting cells with miR-365-3p mimic which effectively rescued the endogenous level of the microRNA (**Supplementary Figure 5**). While IL-17 stimulated dramatic increase of two representative pro-inflammatory cytokines, IL-6 and KC (official symbol Cxcl1) as shown in qRT-PCR analysis (**Figure 4A**), rescue of miR-365-3p level could significantly reduce the expression of these two cytokines upon IL-17 treatment, suggesting that miR-365-3p exerted negative regulatory activity on the pro-inflammatory effects of IL-17 in MLE12 cells. Additionally, the negating role of miR-365-3p was also found in mouse monocyte macrophage J774a.1 as well. Increased IL-6 expression of IL-17-stimulated J774a.1 cells was tuned down to the baseline upon addition of miR-365-3p mimic (**Figure 4B**).

### miR-365-3p also serves as negative regulator in IL-17 induced inflammatory response in human airway epithelial cells and macrophages

We then wondered whether miR-365-3p is also negative regulator of IL-17 in human airway cells. By comparing human and murine microRNA sequences using the miRBase database, miR-365-3p was found to be conservatively expressed in both human and mouse, sharing identical sequence (**Figure 5A**). has-miR-365a-3p, the human homologue of murine miR-365-3p, has been reported to be downregulated in severe asthmatic patients (20, 21), necessitating further examination of its role in IL-17 dominated human asthmatic inflammation response. As expected, human IL-17 (hIL-17) was effective to promote expression of IL-8 (official symbol CXCL8) and IL-6 in human lung epithelial A549 cells by ~2 folds (P<0.01) and ~1.5 folds (P<0.05), respectively, while rescue of endogenous of miR-365-3p almost abolished the IL-17-elevated IL-8 and IL-6 expression (**Figure 5B**). The negative regulatory effect of miR-365-3p on pro-inflammatory effects of human IL-17 was also verified in human macrophages. Human monocyte U937 was differentiated into macrophages with 200 ng/ml PMA for three days, followed by treatment with hIL-17, which in turn stimulated expression of IL-6. As expected, the supplementation of miR-365-3p mimic negated the increase of IL-6 induced by IL-17 treatment (**Figure 5C**). Taken together, the data obtained on human cells indicated that miR-365-3p also served as a negative regulator in IL17-mediated inflammation in human airway cells.

### ARRB2 is the key mediator of miR-365-3p negated proinflammatory effect of IL-17

To further illustrate the mechanisms how miR-365-3p downregulated IL-17-mediated inflammatory response, we firstly examined the activation of NF-kB pathway, the major signaling pathway of IL-17 and found that phosphorylation level of p65, the key player of Nf-κB pathway did not change in miR-365-3p-transfected cells in comparison with mock-treated control cells (**Supplementary Figure 6**), indicating that NF-κB signaling pathway was not involved in the negative regulatory function of miR-365-3p on IL-17 signaling. We then used Targetscan database to predict the potential target genes of miR-365-3p. Among these predicted target genes, critical transcription factors such as Rel, Ets1, Bcl11b, Prdm1, Maf, and Ikzf4, and asthma-related molecules including TRAF3, ARRB2, Sgk1, PIK3R3, ADAM10, ADM Pten and Nr3c1 are the ones that have been reported in PubMed to be involved in pulmonary inflammation response of asthma (**Figure 6A**), whose expression were subsequently examined in mouse lung tissue (**Supplementary Figure 7**). The qRT-PCR results showed that the expression of ARRB2, the essential regulator of GPCR signaling pathway, was significantly higher in wildtype asthmatic group than control group, while IL-17 deficiency diminished its responsiveness to HDM stimulation (**Figures 6B** and **Supplementary Figure 7**), indicating its involvement of IL-17-mediated pathogenesis.

To validate whether ARRB2 was directly targeted by miR-365-3p, luciferase reporter assay constructed with ARRB2 3’UTR was performed. As shown in **Figure 6C**, the addition of miR-365-3p mimic reduced the luciferase activity to ~50% of the original level, suggesting mRNA of ARRB2 gene was directed targeted by miR-365-3p, in consistence with the following qRT-PCR and immunoblotting analysis that showed miR-365-3p mimic reduced the expression of ARRB2 in MLE-12 cells at both mRNA and protein level (**Figures 6D, E**). The regulatory effect of miR-365-3p on ARRB2 expression was further examined in murine lung tissue (**Supplementary Figure 8**) and human airway cells. As shown in **Figure 6F**, while hIL-17 elevated the expression of ARRB2, miR-365-3p supplementation significantly decreased the expression of ARRB2, validating the existence of regulatory relationship between miR-365-3p and ARRB2 in human airway cells. These data, convincingly, demonstrated that ARRB2 was one of direct targets of miR-365-3p in both murine and human asthmatic pathogenesis.

We then, logically, investigated whether ARRB2 was the effector of miR-365-3p to negate IL-17-provoked inflammation. To validate the mediator role of ARRB2, its expression level was manipulated by RNA interfering (RNAi) or overexpressing plasmid in MLE-12 cells. Cytokines production from MLE-12 cells transfected with either ARRB2-siRNA or ARRB2-expressing pcDNA3.1 plasmid was quantified by qRT-PCR. As the result shown in **Figures 6G–J**, the expression of KC was significantly reduced in ARRB2-knockdown cells and elevated in ARRB2-overexpression cells.

**Figure 7:**
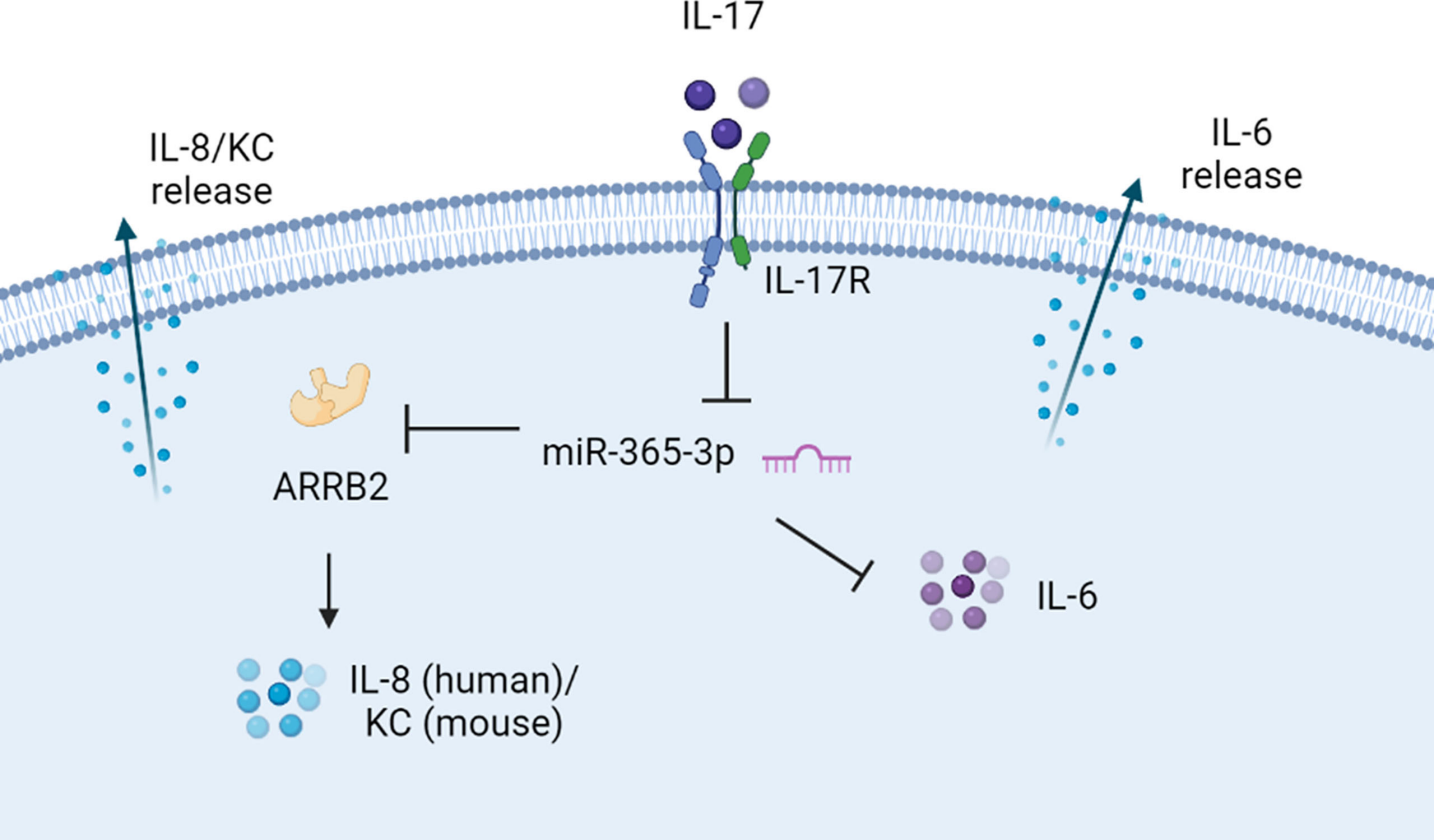
The proposed scheme of negative regulating role of miR-365-3p in IL-17 induced KC and IL-6 production. miR-365-3p, which was diminished by IL-17 in murine and human asthmatic pathogenesis, functioned as an essential negative mediator to suppress IL8 (KC for mouse) and IL-6 upon IL-17 stimulation by targeting ARRB2.

Collectively, our data supported the notion that miR-365-3p, which was diminished by IL-17 in murine and human asthmatic pathogenesis, functioned as an essential negative mediator in IL-17-stimuated inflammatory response by targeting ARRB2 (**Figure 7**).

## Discussion

Previous studies have unraveled the role of IL-17 in provoking the release of pro-inflammatory cytokines and chemokines such as IL-6, TNF-α, IL-1β, CXCL1, IL-8 and MCP-1 (22) in various inflammatory diseases, of which asthma was highlighted since IL-17-orchestrated IL-8 synthesis is crucial to promote neutrophil recruitment and disrupt neutrophil homeostasis in severe and fatal asthma (23). Consistent with previous animal model studies, the asthmatic animal model established in our study using HDM extract induction verified the pivotal role of IL-17 to o maintain the inflammatory phenotype of asthma (24, 25). Nonetheless, due to the different types of allergen and mice used in our asthmatic model, the results obtained in this study slightly differed from the previous murine models. As shown in our results, IL-17 deficiency prohibited neutrophil infiltration rather than eosinophil infiltration in lung tissue and reduces level of KC in BALF, supporting the dominant role of IL-17 in recruiting neutrophils. Furthermore, in our models, IL-17 deficiency did not diminish HDM-induced airway hyper-responsiveness (AHR) (data not shown), which is different from the promoting role of IL-17 in asthmatic airway hyper-responsiveness in previous reports (26).

Although deficiency of Th2 cytokines is believed to promote IL-17 expression in human and IL-17 mediated inflammation in mice (27, 28), severe asthmatic patients frequently display mixed patterns of inflammation including Th2-induced eosinophils and Th17-induced neutrophils (29). Our results, nonetheless, revealed that IL-17 deficiency was also effective to reduce the production of Th-2 cytokines such as IL-4, IL-5, and IL-13, implying a synergistic interaction between Th2 and Th17 responses. However, IL-17 deficiency did not reduce the eosinophil recruitment in the asthmatic mouse model. Given that anti-IL5 therapy had limited impact on eosinophil recruitment and asthma exacerbations (30, 31), there might be a potential alternative mechanism of eosinophil infiltration.

To our knowledge, this is the first report describing a link between miR-365-3p and IL-17 in asthma. A total of 31 miRNAs were dysregulated in WT HDM mice compared with WT control group. Among these altered miRNAs, miR-155-5p (32), miR-148a-3p (14), miR-494-3p (33), miR-142-3p (34), miR-378d (35), miR-181c-5p (36), miR-21a-5p (37), miR-3107-5p (38) and miR-133a-3p (39) have been reported to be involved in asthma, but none of them were associated with IL-17 activity in our analysis. Nevertheless, we found miR-365-3p is negatively regulated by IL-17 in HDM-induced asthma mice, which coincided with the observations in human asthma patients demonstrating that human homologue of murine miR-365-3p, hsa-miR-365a-3p, was negatively correlated with severity of asthma and serum IL-17 level (20, 21). And reasonably, miR-365-3p has the potential to be a biomarker for the IL17-dominated asthmatic inflammation.

Whether the downregulated miR-365-3p was active participator or just bystander in IL-17-mediated inflammation was examined by manipulating its level through introducing exogeneous miR-365-3p mimics. Our results confirmed that miR-365-3p actively antagonized IL-17-mediated inflammatory cytokine elevation from airway epithelial cells and macrophages, not only in murine cells but also in human cells, revealing the universal negating effect of miR-365-3p against IL-17 across the two species. Although various pathways including p38, Erk, NF-κB and PI3K/Akt-dependent pathways have been reported to be involved in the pro-inflammatory activity of IL-17 in previous studies (40, 41), the participation and possible role of microRNA in IL-17-initiated asthmatic inflammation has been barely referred. Our current finding, for the first time, established the correlation between IL-17 and miRNA in asthmatic pathogenesis. Nonetheless, since role of miRNAs has been well recognized in asthma, our current finding added a new expected piece to the puzzle, reinforcing a recent study that miRNA was indispensable for IL-17 to induce secretion of inflammatory factors and chemokines by astrocytes in experimental autoimmune encephalomyelitis (16).

To further illustrate the downstream targets of miR-365-3p to negate IL-17-mediated asthmatic inflammation, ARRB2 (beta-arrestin-2), an essential regulator of G protein-coupled receptor signaling, was selected out in the lung tissue of diseased animals. ARRB2, well known to regulate G protein-coupled receptor function and receptor internalization (42), has been also recognized as an important pro-inflammatory mediator by promoting CD4^+^ T cells migration, Th2 cell chemotaxis, and inflammatory cytokine production in asthma (43, 44). Increased ARRB2 expression in OVA (ovalbumin)-challenged mice and diminished inflammatory responses in ARRB2 deficiency mice (45) implied the key role of ARRB2 in the pathogenesis of asthma. Consistently, our study reinforced the essential regulatory role of ARRB2, as the target of miR-365-3p, in the inflammatory response in IL-17-dependent asthma pathogenesis.

Although both miR-365-3p and ARRB2 have been reported to exert the regulatory roles in cytokine production individually (46, 47), this study provided the first clue to connect these two regulators together in IL-17-mediated asthmatic airway inflammation and proposed the possible IL-7/miR-365-3p/ARRB2/KC(IL-8) axis in asthmatic inflammation (**Figure 7**). It was also noted that alteration of ARRB2 expression did not influence IL-6 synthesis upon IL-17 stimulation. Given the previous study indicating that miR-365-3p could directly target IL-6 mRNA for degradation (48), ARRB2 may not participate in the regulation of IL-6.

In summary, through miRNAs expression profiling in wildtype and IL-17KO chronic asthmatic mouse models, we established the first connection between miR-365-3p and IL-17 in asthmatic pathogenesis and elucidated the possible mechanisms thereof, whereas more details remain to be further explored. This current study provides a new perspective to understand the pathological role of miRNA in IL-17-mediated asthmatic inflammation and may inspire new therapeutic strategies to alleviate neutrophilic airway inflammation in asthmatic pathogenesis.

## Conclusions

Taken together, these findings suggest that IL-17 plays an important role in the inflammatory response and airway remodeling, and miR-365-3p may be a key miRNA involved in this regulation.

## Data availability statement

The data that support the findings of this study are available from the corresponding authors upon request. The miRNA microarray data presented in the study are deposited in the GEO repository, accession number GSE207659. Website Address: https://www.ncbi.nlm.nih.gov/geo/query/acc.cgi?acc=GSE207659.

## Ethics statement

The animal study was reviewed and approved by Animal Care Committee of McGill University.

## Author contributions

WW and JF performed qRT-PCR, western blotting and analyzed the results. YL coordinated the animal experiments. XQ cultured the cells, DS coordinated the histochemistry staining. QQ coordinated the animal experiments. TX did the data analysis. QH revised the manuscript. XD performed ELISA. DX and YC designed the study, performed the animal experiments, and drafted the manuscript. All authors contributed to the article and approved the submitted version.

## Funding

This study was supported by the National Natural Science Foundation of China (32070134 to DX and 31501044 to YC), Natural Science Basic Research Plan in Shaanxi Province of China (2020JM-002), International Cooperation, Exchange Program of Science and Technology of Shaanxi Province (2020KW-046), and The Science and Technology Innovation Base-Open and Sharing Platform of Science and Technology Resource Project of Shaanxi Province (2019PT-26).

## Acknowledgments

We thank H. Li from School of Life Science and Technology, Xi’an Jiaotong University and Instrument Analysis Center of Xi’an Jiaotong University for their (Z. Ren and Y. Chen) technical assistance.

## Conflict of interest

The authors declare that the research was conducted in the absence of any commercial or financial relationships that could be construed as a potential conflict of interest.

## Publisher’s note

All claims expressed in this article are solely those of the authors and do not necessarily represent those of their affiliated organizations, or those of the publisher, the editors and the reviewers. Any product that may be evaluated in this article, or claim that may be made by its manufacturer, is not guaranteed or endorsed by the publisher.

## Abbreviations

IL, interleukin; HDM, house dust mite; KO knock out; BALF bronchoalveolar lavage fluid; qRT-PCR quantitative reverse transcriptase polymerase chain reaction; WT wild type; AHR airway hyper responsiveness; KC keratinocyte chemoattractant; ARRB2 beta arrestin 2; EAE experimental autoimmune encephalitis; RA rheumatoid arthritis; CXCL C-X-C Motif Chemokine Ligand; ASMC airway smooth muscle cells; PBS phosphate-buffered saline; TNF-α tumor necrosis factor-α; IFN-γ interferon-γ; α-SMA α-smooth muscle actin; FASL Fas-ligand; TRAF3 TNF Receptor Associated Factor 3; Sgk1 Serum/Glucocorticoid Regulated Kinase 1; PIK3R3 phosphoinositide-3-kinase regulatory subunit 3; ADAM10 ADAM metallopeptidase domain 10; ADM Adrenomedullin; DMEM Dulbecco’s modified Eagle’s medium; HEPES N-2-hydroxyethylpiperazine-N_-2-ethanesulfonic acid; HITES hydrocortisone, insulin, transferrin, estrogen, and selenium; UTR untranslated region; MLE-12 Murine Lung Epithelial-12; NC negative control; SDS sodium dodecyl sulfate; PAGE polyacrylamide gel electrophoresis; PVDF polyvinylidene difluoride; BSA bovine serum albumin; TBS Tris-buffered saline; HRP horseradish peroxidase; ECL enhanced chemiluminescence; ELISA enzyme-linked immunosorbent assay; OVA ovalbumin.
