# Supplementary material for: MiR-365-3p is a negative regulator in IL-17-mediated asthmatic inflammation
Source: Front Immunol. 2022 Jul 26;13:953714. doi: 10.3389/fimmu.2022.953714 (PMC9361323; doi:10.3389/fimmu.2022.953714)

**Supplementary Table 1.** The sequence of primers for real-time PCR.

| **Gene** | **Sequence (5’-3’)** | **Direction** |
| --- | --- | --- |
| Pten | AACAAAGACAAGGCCAACCG | Forward |
|  | TGGATCAGAGTCAGTGGTGTC | Reverse |
| Nr3c1- | TGTGAGTTCTCCTCCGTCCA | Forward |
|  | GGTAATTGTGCTGTCCTTCCAC | Reverse |
| Rel | CGAGATTGTGAGGGTGAGGC | Forward |
|  | GTTACGAATGCGAGGGACGAT | Reverse |
| Ets1 | TCGGAAGAACTCCTGTCCCT | Forward |
|  | AGAAACTGCCACAGCTGGAT | Reverse |
| Bcl11b | CACCCCCGACGAAGATGACCAC | Forward |
|  | CGGCCCGGGCTCCAGGTAGATG | Reverse |
| Prdm1 | CCGTAGAAAAGGAGGGACCG | Forward |
|  | CAAGGTCGTACCCACACGTT | Reverse |
| Maf | GAAAGGGACGCCTACAAGGAG | Forward |
|  | CCACGGAGCATTTAACAAGGT | Reverse |
| Ikzf4 | CGATGAGGAGTCGAGCAGAC | Forward |
|  | CCATCGCAGTAGCCTAAGGG | Reverse |

**Supplementary Figure 1.** H&E staining of mouse lung tissue sections. Scale bars=100μm.


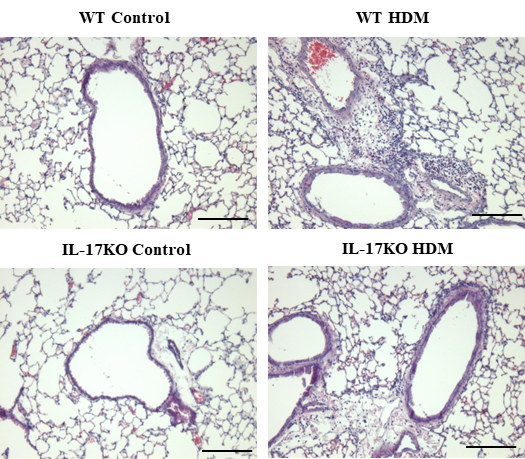


**Supplementary Figure 2.** Percentages of inflammatory cells in total BALF cells of mouse model. N=10.


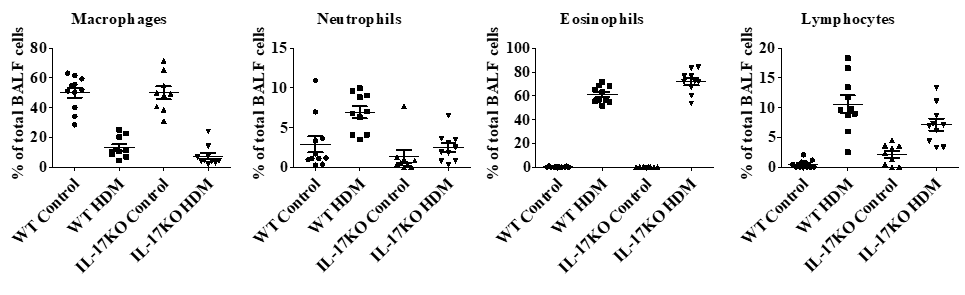


**Supplementary Figure 3.** The expression of IFN-gamma in BALF of mouse model. *P<0.05, N=10.

**Supplementary Figure 4.** Expression of miRNAs selected from microRNA array in mouse lung tissue. The expression of miRNAs was verified by qRT-PCR. ^*^P<0.05, ^**^P<0.01, ***P<0.001. N=10.

**
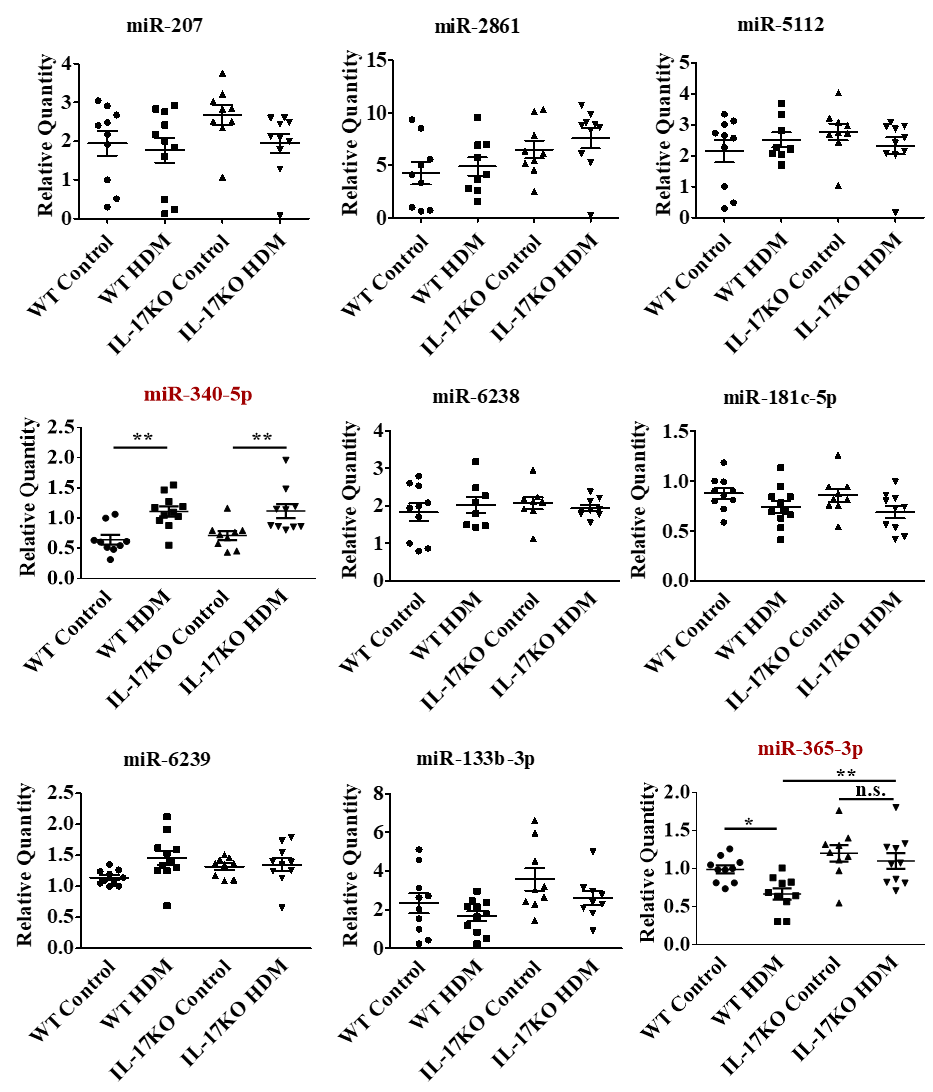
**

**Supplementary Figure 5.** The level of miR-365-3p in transfected MLE-12 cells. N=3.

**Supplementary Figure 6.** Western blot of p-P65 for NF-kB signal pathway in miR-365-3p transfected MLE-12 cells.


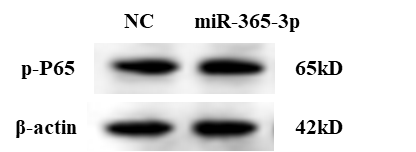


**Supplementary Figure 7.** QRT-PCR validation of target gene expression in asthmatic mouse model. N=10


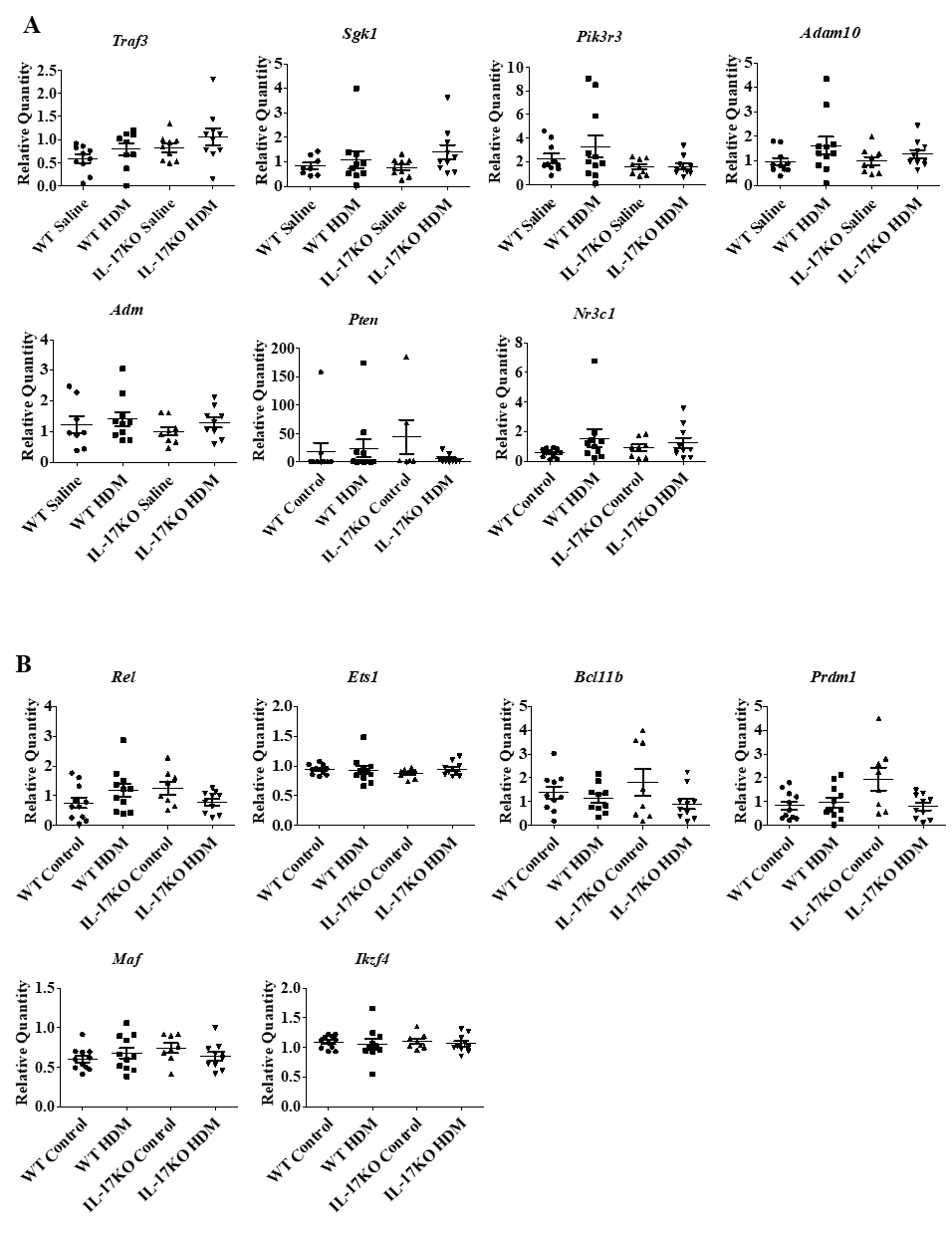


**Supplementary Figure 8.** The expression of ARRB2 was detected by western-blot.


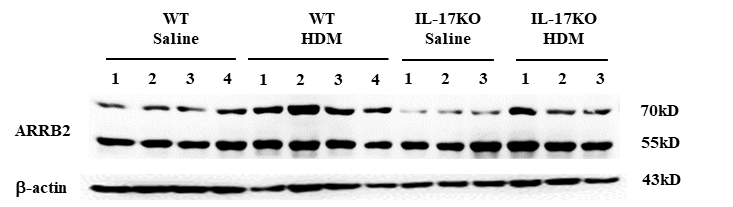

Supplement: Supplementary file 1 [file DataSheet_1.docx]
